# Age and moral disgust: An experimental priming effects vignette study

**DOI:** 10.1371/journal.pone.0295039

**Published:** 2024-02-13

**Authors:** Guido Corradi, Pilar Aguilar, Fernando Aguiar, Antonio Olivera-La Rosa

**Affiliations:** 1 Departamento de Psicología, Facultad de Salud, Universidad Camilo José Cela, Madrid, Spain; 2 BEATLES Research Group, University of Balearic Islands, Illes Balears, Spain; 3 Department of Social Psychology, College of Psychology, Universidad de Sevilla, Sevilla, Spain; 4 Institute of Philosophy, CSIC, Madrid, Spain; 5 Department of Psychology and Social Sciences, Universidad Católica Luis Amigó, Medellín, Colombia; 6 Human Evolution and Cognition Group, Associated Group to IFISC (University of the Balearic Islands – CSIC), Palma de Mallorca, Spain; University of Amsterdam, NETHERLANDS

## 1. Description (optional)

The latest advances in the field suggest that disgust is involved in certain aspects of moral psychology [1–3]. Also, people are known to judge the actions of old people differently to those of young people [4, 5]. Previous work in a vignette study and correlational research design [6] has found that disgust sensitivity affects how harshly moral violations of old adults are judged. However, evidence on the relationship between disgust sensitivity and moral judgments (i.e., judgments of a moral violation) towards the elderly is still scarce and requires further experimental studies. Building on the reviewed literature, the present research is intended to extend current research in this largely unexplored topic, by examining whether the influence of affective priming of disgusting pictures on moral judgments is sensitive to the target’s age.

## 2. Hypotheses (required)

The study of the cognitive/affective foundations of stigmatization indicates that disgust is a relevant variable in this process [3, 7, 8]. Disgust is at its core an emotional response that is elicited by cues of parasites and disease, experienced as a sense of revulsion, offense, notions of pollution, and a behavioral tendency to avoid a diverse pool of stimuli [9, 10]. Disgust sensitivity (i.e., the degree to which people experience disgust) varies remarkably across individuals, cultures, and geographical regions [11–13]. There is evidence that disgust sensitivity decreases with age [14] and that women tend to be more sensitive to disgust than men [12, 15]. Disgust sensitivity is associated with several areas of psychology, such as personality traits, psychiatric disorders, and social belief systems [16, 17].

Although it is widely held that disgust evolved as a pathogen avoidance mechanism [18, 19], some authors argued that disgust extended its original function to also protect the psyche from moral degradation [20]. Several studies suggest that disgust is involved in certain aspects of moral cognition [1–3]. Disgust can exert a causal role in moral judgments (i.e., judgments of a moral violation), can be a consequence of moral violations, or can “moralize” otherwise neutral acts and persons [21, 22]. For instance, some studies found that disgust experienced incidentally makes moral judgments more harsh [23, 24], although this influence may be susceptible to certain moderators [25, 26], such as individual sensitivity to bodily cues [27]. Nevertheless, the role of incidental disgust in moral judgments remains controversial and requires further research [28, 29].

Some authors claim that disgust triggered by a subclass of egregious moral violations (e.g. enjoyment of child pornography) can "degrade" the perpetrator, functioning as a guard against such actions being carried out [20]. Giner-Sorolla and colleagues [3] suggest that disgust not only helps people to value what is "good" or what is "rotten" socially, but also informs perceived intentions and characteristics of the social actors, especially judgments of moral character. Since the disgusting is perceived as degraded and polluting [30], disgust establishes an implicit order in which the target of the repugnant is regarded as inferior and impure [7]. Consistent with this, disgust influences negative attitudes to a variety of outgroups, such as immigrants, foreigners, socially deviant groups [8, 31], obese people [32], and gay men [33, 34]. Disgust, then, is an emotion with a great capacity to stigmatize, i.e. rejecting not just individuals but entire social groups [35, 36].

One social group that is often included in the realm of disgust is older adults [5, 37]. Some evidence suggests that older adults do not necessarily provoke disgust by themselves, but as representatives of old age per se [38]. That is, disgust towards old adults may be explained because of biases about aging as a whole. Perception of vulnerability to disease and awareness of ageing may contribute to this negative bias [39]. Thus, old age is rejected as much as it could transmit illnesses and, what is more, remind us of our mortality [40–42]. Although ageism (i.e., negative attitudes towards older adults) is pervasive in many societies, more research is needed on its correlates and predictors [43]. A consistent finding is that males are more ageist than females [44, 45]. Ageing anxiety (anxiety about getting older) has been related to ageism in several studies [44, 46, 47, but see [48]]. Some authors claim that ageism in younger people should be explained as part of a coping strategy to deal with unpleasant thoughts of mortality [49].

Despite anecdotal evidence supporting this link, empirical evidence on disgust towards old adults remains limited and offers mixed results. Nicol and his colleagues [43] found that prejudice towards older adults is related to disgust sensitivity, but that this relationship is mediated by perceived vulnerability to disease and ageing anxiety. A recent study [6] found that older adults were judged less morally harshly than younger adults, with higher disgust sensitivity being associated with harsher moral judgments, irrespective of the age of the perpetrator. Interestingly, lower disgust sensitivity was associated with less harsh (less severe) moral judgments towards older people than to younger people, (e.g.) suggesting that low disgust sensitivity could partially underlie compassionate paternalism towards the elderly. Altogether, actual empirical research on the role of disgust in moral judgments toward the elderly suggest that this relationship may be more complex (e.g, sensitive to moderators) than predicted by theoretical assumptions and anecdotal evidence.

The fact that the influence of incidental affective responses on moral judgments appears to be largely automatic [50, 51] encourages affective priming as a relevant tool for the study of moral cognition. This experimental paradigm is built on the assumption that the affective nature of the prime (e.g. a disgusting image) will impact the subject’s evaluation of the target stimulus (e.g. a moral dilemma). Indeed, affective priming often occurs independently of evaluative intention and of awareness of the prime [52]. Although affective priming has been effectively applied to a vast number of studies [53], this technique has scarcely been applied to moral research. There is evidence that erotic pictures presented in a suboptimal manner (but not other types of pleasant pictures) increase utilitarian moral judgments, which might be due to the erotic stimuli being more arousing [54]. Affective priming through disgusting pictures (depicting human mutilation) exclusively reduced the severity of moral judgments (with no effect on non-moral judgments [55]). The effect of disgust priming on moral judgments may be moderated by how sensitive the individual is to disgust: while disgusting pictures favoured utilitarian judgments in participants with higher disgust sensitivity, the same pictures favoured deontological judgments in participants with lower disgust sensitivity [56]. In sum, the few studies conducted on the effects of affective priming on moral judgments show mixed results, suggesting that more research is needed to disambiguate the direction of the effects of affective priming on the harshness of moral judgments. For instance, whether affective priming of disgusting pictures would have a different effect on the harshness of moral judgments than other types of affective pictures (i.e, depicting stimuli associated with different emotional responses) and whether this effect varies depending on the age of the person performing the action requires further research.

Building on the reviewed literature, the present research aims to conceptually replicate and extend Aguiar and his colleagues’ [6] finding that disgust sensitivity responds to the target’s age in the making of moral judgments, by examining whether the influence of affective priming of disgusting pictures on moral judgments is sensitive to the target’s age. Consequently, we propose the following research questions (RQ) summarized in the following table.

**Table:** 

RQ	Explanation	Design decision	Statistical tests and expectations
1	To investigate the role of disgust sensitivity in judging two age scenarios (characterized by old or young actors) more or less morally harshly.	Manipulation between participants is actors`age (young vs old).	We expect that higher disgust sensitivity is related to harsher (i.e., more severe) moral judgments. We expect the relation between disgust sensitivity and harshness of moral judgments to be stronger in the old aged vignettes relative to young ones. We expect the less-disgust sensitive participants will judge the old themed vignettes less morally harsh than young actor vignettes. Highly disgust-sensitive participants will evidence harsher moral judgments, regardless of the age of the vignette actor
2	Investigate the effect of affective pictures (primes) on the harshness of moral judgments	Within participants manipulation with affective pictures (brief emotionally valenced images with sad, neutral and disgust primes).	We expect that disgust pictures will have a different effect on the harshness of moral judgments than other affective pictures We made no prediction about the direction of the relationship, as explained above, the literature on affective priming and moral judgments could support either a positive or negative difference

## 3. Design plan

### 1. Study type (required)

Experimental: participants are randomly assigned to one of two experimental conditions following a between participants design. Moreover, within this there is an experimental condition with randomization of trial order. Correlational design: there are measures (ageism scores, emotions elicited, etc.) which are only measured, but not manipulated.

### 2. Blinding (required)

Participants will be blind regarding the condition (old or young themed vignettes) to which they are assigned to. This decision is taken to reduce possible effects of socially desirable responses if participants figure the old and young comparison. We consider that keeping target age within-subjects would make obvious the experimental manipulation. This is because moral vignettes in both conditions (Old vs Young) only differ in the age of the target. For experimental purposes, it was crucial that participants were blind to the condition. Therefore, we decided to manipulate target age between-subjects.

### 3. Is there any additional blinding in this study?

There is no additional blinding.

### 4. Study design (required)

This study involved a 2 x 3 mixed design. On one hand we have a between-subject research design in which participants are randomly assigned to one of the two conditions: The Old Condition, and the Young Condition. Conditions refer to the same vignettes but the main character of the vignette is an old or young person. On the other hand we have a within-subject research design in which 24 affective primes (8 sad, 8 disgust, and 8 neutral control stimuli) will be shown briefly (for 16ms). The dependent variable is the harshness (i.e., severity) of moral judgments (i.e., judgments of a moral violation). Specifically, participants judged how acceptable each behavior depicted in the vignettes was by using a 1 to 7 Likert style scale (“How acceptable do you think this behavior is?”; as Laakasuo et Al. [57] did). Lower score on this item reflects harsher judgments. The participants were instructed to carefully read the vignette displayed and then to press the keyboard bar. Once the keyboard bar is pressed the affective prime (the picture) is shown for 16ms and after that a 100ms white noise mask is displayed. After that, the dependent variable is presented with the keyboard numbers 1–7. Once the response is given, a black screen appears while the next trial is being prepared. Participants were explicitly instructed to look at the screen continuously. Also, three examples are displayed before the test as practice. At the end of the study socio-demographic information and measures about ageism, disgust sensitivity, and emotions regarding young and old people are asked for.

### 5. Randomization (optional)

We randomize the participants by getting a random number from a *JavaScript* code included in the experimental software OpenSesame Online, which allocates to half the participants the number 0 which assigns them to the “Young” condition, and to the other half the number 1, assigning them to the “Old” condition.

The trials with the 24 vignettes are presented in a random order. There are four types of vignettes (fairness, care, authority, and sanctity) and three types of prime images (neutral, sad, and disgust ones). We pair the primes and the vignette types, accounting for the same proportion of prime types in each vignette type. This randomization is created by the *JavaScript* code.

### 6. Sampling plan

We plan to gather 250 participants with ages ranging from 28 to 55 years from Prolific^™^, a platform for running online studies. We are targeting participants from Spain whose first language is Spanish. We aimed to sample middle-aged participants to weaken a possible effect of in-group bias [6, 40].

### 7. Existing data (required)

The data have not yet been collected, created, or realised. However, a pilot study was conducted to determine plausible parameters for the power analysis conducted. Raw data, analysis, and results are available in the project’s OSF link. The project and the experimental protocol were approved by the Research Ethics Committee’s Ethical Committee at the Virgen del Rocío University Hospital (Seville, Spain) prior to data collection.

### 8. Explanation of existing data (optional)

In order to calculate some parameters for simulations required for the power plan of the study as well as to test the online platform for data collection, we recruited 41 participants from Prolific^™^.

We report the descriptive statistics for the main dependent variable, scales, and sociodemographic information. Besides sadness priming there are no relevant differences in the means for the harshness assessments (see Table 1).

**Table 1:** Descriptive statistics of main variables of interest by condition.

Moral judgment	Old Condition *M (SD)*	Young Condition *M (SD)*
Condition Age	2.91 (0.73)	2.54 (0.54)
Prime Disgust	2.85 (0.90)	2.71 (0.65)
Prime Sadness	3.05 (0.70)	2.53 (0.55)
Prime Neutral	2.85 (0.90)	2.39 (0.53)
Scales	Old Condition M (SD)	Young Condition M (SD)
Disgust Sensitivity Score	2.30 (0.40)	2.43 (0.45)
Ageism Scale Score	2.85 (0.17)	2.79 (0.22)
Sociodemographic information	Old Condition M (SD) / %	Young Condition M (SD) / %
Participant’s Age in years	33.8 (10.4)	31.9 (10.4)
Sex: male	52%	60%

Note: Lower scores indicate harsher moral judgment

For inferential purposes we modeled the response (harshness moral judgment) with a linear mixed model effects regression. In the model we included the condition interaction (Young vs Old) with the disgust sensitivity score and condition interaction with prime type. As random effects we included participant and vignette identifiers which produced a convergent model. Regarding the inferential model, we found that the convergent model included item and participant as random effect (see Table 2). Judging by the intraclass correlation coefficient value the mixed model seems to be an adequate model for the data presented. Coefficients show a small positive effect of the Old Condition and a large negative effect with the interaction between disgust sensitivity and condition age. Priming conditions resulted in modest coefficient values.

**Table 2:** Coefficients of model for testing H1 and H2 on the pilot data.

Coefficient	*B* 95% CI *β*	*p* value
Fixed effects		
Condition Age [Old]	0.22 [-0.22, 0.66] 0.13	0.325
Disgust Sensitivity Score	-0.06 [-0.83, 0.70] -0.01	0.872
Condition Age x Disgust Sensitivity Score	-1.32 [-2.42, -0.22] -0.27	0.019
Prime [Sadness]	0.30 [-0.71, 1.30] 0.18	0.561
Prime [Disgust]	0.21 [-0.79, 1.22] 0.13	0.674
Condition Age x Prime [Sadness]	-0.14 [-0.54, 0.25] -0.09	0.471
Condition Age x Prime [Disgust]	-0.12 [-0.51, 0.27] -0,07	0.530
Random Effects variance components	*σ* 95% CI	
Participant	0.56 [0.43, 0.74] 0.33	
Item	0.98 [0.72, 1.34] 0.58	61
Residual	1.26 [1.20, 1.32]	
Model adjustment indices		
ICC	0.49	
R^2^ Conditional (Fixed and random effects)	0.47	
R^2^ Marginal (Fixed effects)	0.04	

### 9. Data collection procedures (required)

Data will be collected by means of a Prolific^™^ sample for which we will pay the £9.00/hr rate. We will use the online platform JATOS [58] deploying Opensesame Online instance [59].

### 10. Sample size (required)

**Fig 1:**
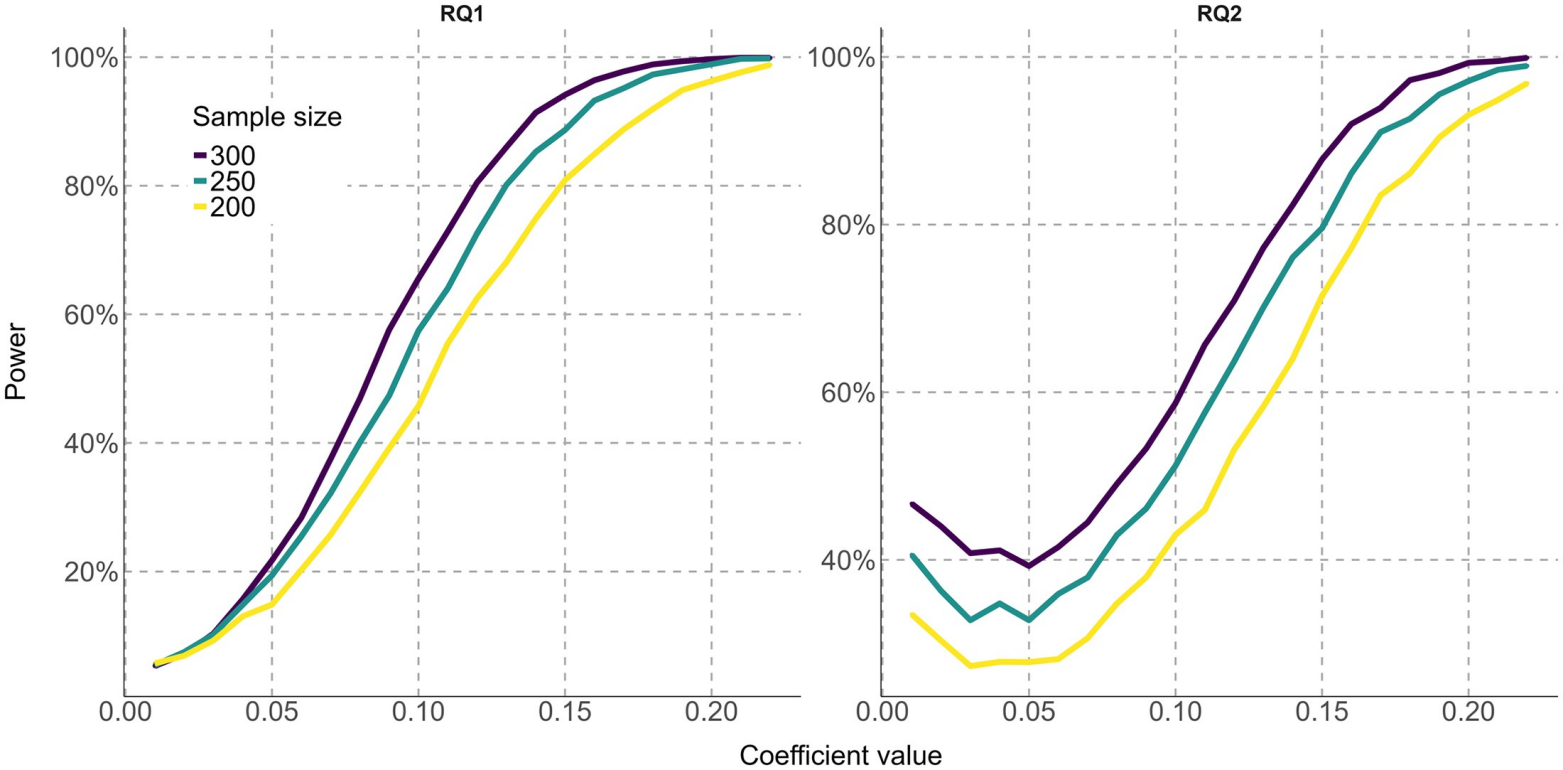
Power curves for RQ1 and RQ2 by sample size. Note: Coefficients are shown in absolute values.

Our target sample size is 250 participants, which is the approximate maximum capacity covered by the research project’s budget for participants recruiting. In order to interpret future results, we performed a power sensitivity analysis regarding RQ1 (i.e. condition age by disgust sensitivity) and RQ2 (i.e. condition age by prime type). Our analytic strategy consists in simulating different effect sizes (standardized regression coefficient in our case) in our model and estimate the power related to that sample and effect. We estimated the model to test RQ1 and RQ2 with the pilot data. With this model we estimated the correlation parameters and fixed effects parameters to perform a power sensitivity analysis. By means of 5000 simulations using *simr* [60] we changed the standardized regression coefficient (RQ1: 0.01 to 0.40 and RQ2: 0.01 to 0.40) and number of participants (200, 250 and 300) to clarify power and minimum effect sizes detectable by our design given the pilot correlation parameters. We explored different sample sizes to manage possible changes in the available participants. Results show that with our planned sample size (250) we can detect a standardized coefficient of 0.13 for RQ1 and 0.15 for RQ2 (see Fig 1) with 80% power. The data from the pilot study, script for the analysis and tables with results are available at: https://osf.io/mh8w2/?view_only=022da903445f41d29be17cc3cb261f68.

## 4. Variables

### 1. Manipulated variables (optional)

#### Actors´ age

We manipulated the age of the vignette actor (see [6]). Participants were presented with 24 vignettes, six for each moral foundation proposed by Haidt [50]: Care/Harm, Fairness/Cheating, Loyalty/Betrayal, Authority/Subversion and Sanctity/Degradation. These vignettes, describing behavior that violates a specific moral foundation, have already been used in previous studies [6, 61]. In The Old condition, participants were informed that the actor presented in the vignette was an old person whereas, in the Young Condition, the actor of the action was categorized as a young person. We selected the vignettes based on our being able to adapt them to our purposes, that is, if the main character could be substituted by an old or a young character. One example of vignette of the old condition is “You see an old man laughing as he passes a cancer patient with a bald head”. The list is available in the Online Supplementary Repository (https://osf.io/mh8w2/).

#### Priming

We manipulated the type of affective priming by means of briefly presented pictures following the procedure described in Olivera-La Rosa et al [54]. There are three levels of this categorical variable: Disgust Priming, Sad Priming, and Control (neutral) priming. Each priming condition contains 8 stimulus and each of them is presented for 16ms. The 16 stimuli corresponding to disgust and sadness were taken from Binyamin-Suissa et al. [62]. Experiment 1 and the eight neutral pictures were taken from the IAPS [63] picture database. The full list is available in the Online Supplementary Repository (https://osf.io/mh8w2/).

### 2. Measured variables (required)

#### Disgust sensitivity

Participants will complete the 27-item Disgust Scale-Revised-Spanish (DS-RS) [64–66] which is divided into three sections: a “core disgust” section comprising twelve items (measuring food-related disgust, animal-related disgust, and disgust related to body products), an “animal reminder” section comprising eight items (measuring disgust towards death and envelope violations), and a “contamination” section comprising five items (measuring concerns about interpersonal transmission). All items will be rated on 5-point scales (0–4), where 0 = “*Strongly disagree*” and 4 = “*Strongly agree (very true about me)*”. The scale also includes two control-items to identify those participants who are not completing the task properly.

#### Ageism

Stereotypes about aging will be measured among adults using the CENVE Negative Stereotypes Towards Aging Questionnaire [67, 68]. This questionnaire consists of 15 Likert-type questions, ranging from 1 to 4 (1 = “*Strongly disagree”*, 4 = “*Strongly agree”*).

#### Emotions

Participants will be asked to report to what extent a young or an old person, depending on the condition, elicit following emotions related to the moral foundations: Gratitude, Guilt, Anger, Compassion, Pride, Rage, Respect, Fear, and Disgust. The scale responses were provided on a 7-point scale ranging from 1 = “Not at all”, to 5 = “Extremely [50, 69]. For example, in the old condition would be: “Rate from 1 (not at all) to 5 (extremely) the emotions that best reflect your feelings toward the word OLD”.

#### Open response

Participants will be asked about which rationale they think underlie the study with an open response option at the end of the study and if they followed any strategy to answer. Also, any additional comment will be welcomed. Specifically, we will include the following questions: “What do you believe is the purpose of this study?”, “Did you employ any particular strategy when answering the questions?” and “Do you have any other comments?”

## 5. Analysis plan

### 1. Statistical models (required)

In order to handle the different sources of variability present in our data, we will analyse the effects of conditioning on moral judgments and the effects of affective pictures with generalised linear mixed effects models. This method accounts simultaneously for the between-subject and within-subject effects of the independent variables [70]. We will model the maximal random effects structure justified by the design, which in addition to avoiding the loss of power and reducing Type-I error, enhances the possibility of generalising results to other participants and stimuli. However, as noted by Barr et al. [71], when using maximal models, the process of parameter estimation will occasionally fail to produce a solution.

For testing our RQ1 and RQ2, we plan to perform a linear mixed model predicting the main dependent variable of interest (harshness of moral judgment) by the statistical interaction between condition (young vs old) and the disgust sensitivity score, as well as prime type (sad, neutral and disgust pictures) by condition (young vs old) statistical interaction as fixed effects. Participants, items, and primes will be set as random effects. If the model does not converge, we will assess correlation structures to select the cluster to keep. We will assess the coefficient’s statistical significance (with the Satterthwaite method of approximation of degrees of freedom) as well as the confidence interval. As we include some exclusion criteria, we will compare models with and without exclusions to test robustness. Commented script is available in the Online Supplementary Material (https://osf.io/mh8w2/).

### 2. Transformations (optional)

Continuous variables will be mean-centred. We will take “Young condition” as the reference group for Young-Old condition comparisons, for prime type we will take “neutral prime” as the reference group, and “dummy coded” for the sad and disgust ones.

### 3. Inference criteria (optional)

We will use the *p*-value <. 05 criteria. We will use the *parameters*() function of the ‘parameters’ package with the Satterthwaite method of approximation of degrees of freedom to calculate *p*-values. Also, *emmeans*() from the ‘emmeans’ package [72] will be used to create the predicted marginal means, contrasts, and comparisons for fixed effects of models.

### 4. Data exclusion (optional)

We will exclude participants with responses with zero variance (experimental task or questionnaires included) and those which have not included gender or age information, or have more than two missing values in the questionnaire’s responses. We will exclude trials with response time of above 4000ms and participants which have more than 30% trials missing.

We aimed to sample middle-aged participants to weaken a possible effect of in-group bias [40]. Therefore, we will exclude participants aged above 55 and under 28 years.

Based on the control open ended question responses participants who are aware of the experiment’s aim will be excluded from further analysis, also if any participant explicit some response strategy will be assessed it inclusion. We intend to exclude participants who explicitly acknowledge awareness of the experiment’s objectives, that is, those who mention that the study pertains to prejudices about older or younger individuals and/or the impact of images on judgments. Additionally, we plan to exclude participants who report employing strategies that negate the intended effect of the prime(e.g., closing their eyes), or any tactic suggesting that the assessment was not properly conducted (e.g., consistently pressing the same number).

### 5. Missing data (optional)

We will impute the mean for the questionnaire scores if the participant meets the exclusion criteria of more than two missing values.

### 6. Exploratory analysis (optional)

We expect to find that the effects of affective pictures on the harshness of moral judgements will be different in each condition (old vs young actors). We will explore this potential relationship by means of an interaction between prime type and condition.

We expect that certain demographic traits and individual differences may be related to judgment. Therefore, we will look for relationships between demographic variables (age, gender) as well as the control variables (ageism scores, disgust elicited by the word “old”, etc.) and the primary outcome measures of judgements.

Also, we aim to explore the relationship between ageism scores and the emotions elicited by old people as an interaction between the age condition and the harshness of moral judgment using assessment after the experimental phase with scales. We will look at relationships between emotions elicited by old people and the harshness of moral judgements on old themed vignettes. To make these explorations, we will introduce the different predictors into the model and assess the effect by comparing the statistical adjustment (AIC and ANOVA) of each predictor against the model with no predictor included.
